# The role of vaspin as a predictor of coronary angiography result in SCAD (stable coronary artery disease) patients

**DOI:** 10.1186/s12872-017-0550-1

**Published:** 2017-05-08

**Authors:** Matej Stančík, Ivana Ságová, Ema Kantorová, Marián Mokáň

**Affiliations:** 1 Clinic of Internal Medicine I., Jessenius Faculty of Medicine in Martin, Comenius University in Bratislava, Kollárova 2, Martin, 036 59 Slovak Republic; 2 Clinic of Neurology, Jessenius Faculty of Medicine in Martin, Comenius University in Bratislava, Kollárova 2, Martin, 036 59 Slovak Republic

**Keywords:** Adipocytokine, Vaspin, Stable coronary artery disease, Coronary angiography, Pre-test probability

## Abstract

**Background:**

The role of vaspin in the pathogenesis of stable coronary artery disease (SCAD) have been repeatedly addressed in clinical studies. However, from the point of view of clinical practice, the results of earlier studies are still inconclusive.

**Methods:**

The data of 106 SCAD patients who received coronary angiography and 85 coronary artery disease-free controls were collected and analysed. The patients were divided into subgroups according to their pre-test probability (PTP) and according to the result of coronary angiography. Fasting vaspin concentrations were compared between subgroups of SCAD patients and between target group and controls. The effect of age and smoking on the result of coronary angiography was compared to the effect of vaspin using the binomial regression.

**Results:**

We did not find significant difference in vaspin level between target group and controls. Unless the pre-test probability was taken into account, we did not find vaspin difference in the target group, when dividing patients on the basis of presence/absence of significant coronary stenosis. In the subgroup of SCAD patients with PTP between 15% – 65%, those with significant coronary stenoses had higher mean vaspin concentration (0,579 ± 0,898 ng/ml) than patients without significant stenoses. (0,379 ± 0,732 ng/ml) (*t* = −2595; *p* = 0,012; d = 0,658; 1-β = 0,850). Age, smoking status and vaspin significantly contributed to the HSCS prediction in binomial regression model in patients with low PTP (OR: 1.1, 4.9, 8.7, respectively).

**Conclusion:**

According to our results, vaspin cannot be used as an independent marker for the presence of CAD in general population. However, our results indicate that measuring vaspin in SCAD patients might be clinically useful in patients with PTP below 66%.

## Background

Vaspin is recently discovered adipocytokine. For the first time it has been isolated from visceral adipose tissue of rats. The hypothesis that vaspin is an insulin sensitising adipocytokine has been proposed in 2005 [1]. Researchers later described an association of vaspin with obesity and insulin resistance [2]. From the biochemical point of view, vaspin is member of serpin superfamily. It is acting in both paracrine - inside visceral adipose tissue and endocrine manner – affecting mainly central nervous system and liver. In the liver vaspin bounds to GRP 78 chaperone, which has previously been incorporated into cytoplasmic membrane as a response to endoplasmic reticule stress [3]. As a result of this GRP 78 translocation, vaspin bounds to GRP 78 and exerts beneficial effects on endoplasmic reticule stress - induced metabolic dysfunctions. After intrathecal application, vaspin exerted appetite lowering effects which lasted for 24 h and subsequently enhanced insulin sensitivity [4]. This experimental result revealed that vaspin has an effect on central appetite regulation in brain. Moreover, there are indications that vaspin gene mutations could play role in a proportion of genetically determined forms of obesity [5].

The role of vaspin has therefore emerged as an important topic of research in the cardiometabolic field. On a model of aortic intima damage in the setting of diabetes mellitus vaspin, through its bond to GRP 78 – voltage dependent anion channel complex, showed an inhibitory potential on calcium mediated apoptosis and facilitated endothelial reparation [6]. Jung et al. showed positive impact of vaspin towards endothelial nitric oxide synthase activity in endothelium [7]. Based upon these experimental results arose the idea to investigate a connection of vaspin with the degree of coronary artery disease (CAD) lesions in patients suffering from ischaemic heart disease. In the study performed by Kobat et al., vaspin has been significantly lower in patients suffering CAD, when compared to controls [8]. Similarly designed study examining the role of vaspin in patients with unstable angina pectoris, showed significantly lower plasma vaspin concentrations in unstable angina [9]. A recent meta-analysis of six studies studying the connection between vaspin and obesity confirmed higher vaspin concentrations in obese and diabetic patients [10]. Based on the results of these studies, it can be assumed, that higher vaspin is the attendant phenomenon of protective and compensatory mechanisms triggered in the situation of developing cardiometabolic disorder.

On the other hand, Choi et al. published results, which proved the existence of inverse association between vaspin and the level coronary artery stenotic damage represented by Agatston calcium score [11]. However, this association has been found only in the subgroup of women, but not in men. Kadlogou et al. with the cohort of 108 patients suffering from stable coronary artery disease (SCAD) illustrated, that vaspin is independent determinant of CAD severity [12]. Hao et al. recently published paper with results of their own research on the role of vaspin in diabetes mellitus and coronary artery disease. In this study vaspin levels were significantly increased in diabetics compared to healthy individuals and further increased in patients with both diabetes and CAD [13]. Based on the results of these studies we cannot conclude, whether vaspin upregulation exerts protective effects on coronary arteries prior the development of CAD or if vaspin’s action is compensatory only. In connection to the role of vaspin in calcium mediated apoptosis and endothelial reparation, it seems reasonable to assume, that plasma vaspin concentration measurement could be of a great benefit when estimating the probability of hemodynamically significant coronary artery stenosis (HSCS) in a patient suffering from SCAD. However, no clinically usable criteria on vaspin use as a diagnostic marker of HSCS exists nowadays. Further research is needed in order to clarify the role of vaspin concentration measurement in SCAD.

## Study objectives

First objective was to verify the existence of independent relation between vaspin and the diagnosis of SCAD. Second objective was to test the relation between vaspin and the pre–test probability (PTP). Third objective was to address the hypothesis that vaspin has the potential to be used as a marker of HSCS in SCAD patients.

## Methods

### Study population

The study is a double–centre transversal design performed on data gathered from 191 subjects divided into control group and target group. 85 CAD-free controls were enrolled in the occasion of annual preventive medical check-up. Target group consisted of 106 SCAD patients scheduled for elective invasive coronary assessment (ICA), which were performed from April 2015 until December 2016 at our clinic. Study subjects selection was based on inclusion and exclusion criteria distinctly defined for target and control group. Every study subject voluntarily signed an informed consent about study participation.

### Clinical procedures

#### Target group

After a SCAD patient volunteered and signed the informed consent on study participation, we measured waist and hip circumference, height, weight, calculated body mass index (BMI). Each study participant had PTP higher than 15%. ICA was indicated in patients with mid to high probability of cardiovascular event. ICA was also performed in those patients in low cardiovascular event risk, in whom intensified optimal medical therapy (OMT) did not lead to remission of SCAD symptoms. In each study participant estimated PTP was calculated according to ESC 2013 guidelines on diagnostic and treatment of SCAD. There were no patients in the first group, defined as PTP lower than 15%. The second group consisted of patients with PTP 15% - 65%. The third and fourth group was defined by PTP from 66% to 85%, more than 85%, respectively. Transthoracic echocardiography was performed in each study subject. We obtained blood samples in order to perform laboratory tests. Blood samples were taken at basal conditions after night-long fasting, at 7 o’clock in the morning. Selective coronary arteriography (Judkins) was performed at the same day blood samples were taken. Coronary artery stenting was indicated according to criteria defined by 2014 ESC/EACTS guidelines on myocardial revascularisation. Smoking was defined as the presence of habitual smoking at the time of inclusion to the study cohort or the positive anamnesis for habitual smoking in past ten years.

##### Inclusion criteria

SCAD, in each SCAD patient single photon emission tomography and/or cardiac stress test must had affirmed the ischaemic heart disease diagnosis. Only individuals with the evidence of coronary artery disease based on the results of these tests and with objective signs of exertional cardiac ischaemia were enrolled. Each target group subject fulfilled the 2014 ESC/EACTS indication criteria for ICA.

##### Exclusion criteria

Acute forms of ischaemic heart disease, history of myocardial infarction or unstable angina, history of oncological disorder, diabetes mellitus type 1, decompensated heart failure, acute/decompensated endocrinopathies, severe pulmonary arterial hypertension, laboratory or clinical signs suggestive of active infections.

#### Control group

We enrolled 85 CAD-free subjects for the control group. We measured basic biometric parameters, obtained blood samples and performed the identical biochemical analyses as were performed in the target group. Blood samples were taken at basal conditions after night-long fasting, at 7 o’clock in the morning. Smoking definition criteria were the same as in target group.

##### Inclusion criteria

CAD-free individuals free of laboratory or clinical signs of acute illness or subjective symptoms suggestive of acute illness. Each control group subject was enrolled at the occasion of annual preventive medical check–up.

##### Exclusion criteria

Known or suspected CAD, otherwise same as in target group.

### Laboratory procedures

Vaspin level concentrations were measured with commercially available ELISA kits certified for experimental use (Vaspin Human ELISA, EIA-5608, DRG International Inc). Each sample had been obtained in advance of ICA, at the same day ICA was performed and after night-long fasting. Plasma separation (centrifugation: 15 min @ 3500 RPM) was performed immediately after the blood sampling. Plasma samples were deep-frozen at minus 20 degrees Celsius without delay. Deep-frozen plasma samples were stored until the ELISA assay was performed in the certified clinical biochemical laboratory. Each ELISA assay was performed immediately after the sample thawed, repeating the thaw–freezing cycles was strictly forbidden. Each sample has been processed and measured twice. Only the mean values, obtained by double–testing each plasma sample, entered further statistical analyses. In each study participant we performed the battery of standard laboratory tests measuring sodium, potassium, chloride level, creatinine and urea concentration, calculated estimated glomerular filtration rate (eGFR) using the abbreviated Modification of Diet in Renal Disease formula, triglyceride (TG), low-density lipoprotein (LDL), high-density lipoprotein (HDL), total cholesterol (TCH) concentration, C-reactive protein (CRP), liver enzymes, blood count differential and basic coagulation parameters.

### Invasive coronary assessment

Coronary artery angiography was indicated and performed in SCAD patients according to the 2013 ESC Guidelines on the management of SCAD and in accordance to the 2014 ESC/EACTS Guidelines on myocardial revascularisation. Coronary artery stenosis was visually estimated by an experienced interventional cardiologist and was double–checked by another pair of eyes. Proximal LAD and left main stenosis of more than 50% was considered hemodynamically significant. In other coronary vessels, 70% narrowing was considered to be significant. When needed, FFR method was utilized in coronary artery stenting decision-making process. The result of ICA entered our statistical analysis in the form of the binomial HSCS variable (presence/absence of HSCS).

### Statistical methods

Statistical analyses were performed using IBM SPSS Statistics v. 20.0 software. Statistical power was calculated using the G*Power v. 3.0.1 software. In each statistical test performed, the criteria for statistical significance was asymptotic *p*-value (or exact *p*-value where appropriate) ≤ 0,05. Normality testing was performed using Shapiro-Wilk test. In the case of non–normal distribution, we normalised the data using Log10 transformation (positively skewed data) or with the reflection transformation (negatively skewed data). We compared the means of continuous variables using the independent sample t-test. The effect sizes in essential t–statistic tests were expressed as the Cohen’s d-value or coefficient of determination r_bp_^2^. If the homogeneity of variances assumption was not met, we corrected for this violation by adjusting to the degree of freedom using the Welch-Satterthwaite method. Nominal variables were compared using the chi-square test. Correlation analyses were performed using the two-tailed Pearson correlations or point-biserial correlation where appropriate. A Kruskal-Wallis nonparametric test was used to determine if there were statistically significant differences between subgroups of the independent variable on a continuous dependent variable. Binomial regression model was built after exclusion of the cases with studentized residuals higher than 2,5 standard deviations. Box–Tidewell procedure including the Bonferroni correction was performed to test the assumption of linear relation of continuous variables to the logit of the dependent variable.

## Results

Basic characteristics of the target group and control group are compared in Tables 1 and 2. Differences between SCAD subjects divided according to the pre-test probability are highlighted in Tables 3 and 4. In our cohort, there were 69 patients with estimated PTP from 15% to 65%, 33 patients with estimated PTP from 66% to 85% and four patients with PTP more than 85%. Based on this PTP distribution, further statistical analyses were performed after dividing the entire target group into PTP 1 (PTP 15 – 65%) and PTP 2 (PTP more than 65%) subgroup. There were 12 (38,7%) male patients in PTP 2 subgroup in whom HSCS was diagnosed and 13 (34,2%) female patients with HSCS. Out of all 106 patients in the target group, in 55 patients (51,8%) HSCS was diagnosed. In the target group we found higher mean vaspin concentration in women (0,5098 ng/ml ± 0,8883 ng/ml) than in men (0,2624 ng/ml ± 0,4308 ng/ml). This was a statistically significant difference (t(103) = − 2063; *p* = 0,042). A point–biserial correlation between gender and normalised vaspin revealed, that the effect of gender on vaspin concentration is very low (coefficient of determination r_bp_^2^ = 0,0396; *p* = 0,042). The change in vaspin between diabetics and non–diabetics in the target group was non–significant (t(103) = 0,058; *p* = 0,954). Control group did not exhibit significant between-gender difference in vaspin level (t(83) = − 0,954; *p* = 0,343), nor significant vaspin ranks difference (U = 238; z = −1054; *p* = 0,292) between diabetics (mean rank = 34,25) and non–diabetics (mean rank = 43,91). In diabetic SCAD patients we found positive correlation between vaspin and BMI (*r* = 0,431; *p* = 0,022; *n* = 28), vaspin and waist circumference (*r* = 0,447; *p* = 0,017; *n* = 28). In non–diabetic patients we didn’t detect these correlations.

**Table 1:** General characteristics - control group vs. target group

Variable	Control group	Target group	Difference sig. (2-tailed)
Age (years)	59,6 ± 7,7	62,0 ± 8,4	*p* = 0,040
Waist circumference (cm)	103,9 ± 14,6	107,4 ± 11,1	*p* = 0,074
Hip circumference (cm)	110,6 ± 11,9	107 ± 9,6	*P* = 0,018
Waist to hip ratio	0,940 ± 0,085	1002 ± 0,066	*P* < 0,001
Body mass index (kg/m^2^)	30,59 ± 6,01	30,07 ± 4,60	*p* = 0,790
LVEF (%)	59,2 ± 4,4	56,3 ± 7,6	*p* = 0,656
LVEDd (mm)	49,1 ± 3,3	51,4 ± 5,7	*P* = 0,002
IVS (mm)	10,4 ± 1,6	11,3 ± 1,7	*p* = 0,001
LDL (mmol/l)	3,53 ± 0,98	2,61 ± 0,92	*p* < 0,001
HDL (mmol/l)	1,45 ± 0,37	1,32 ± 0,32	*p* = 0,010
TG (mmol/l)	1,80 ± 0,85	1,73 ± 1,02	*p* = 0,315
TCH (mmol/l)	5,69 ± 1,09	4,70 ± 1,09	*p* < 0,001
eGFR (ml/min/1.73m^2^)	92,1 ± 22,1	71,0 ± 15,4	*P* < 0,001
Vaspin (ng/ml)	0,4297 ± 0,6763	0,4212 ± 0,9008	*p* = 0,629

*LVEF* Left ventricular ejection fraction, *LVEDd* left ventricular end-diastolic diameter, *IVS* Interventricular septal thickness, *LDL* Low-density lipoprotein, *HDL* High-density lipoprotein, *TG* Triglyceride, *TCH* Total cholesterol, *eGFR* Estimated glomerular filtration rate

**Table 2:** Nominal variables characteristics - control vs. target group

Variable	Control group	Target group	Difference sig. (2-tailed)
Gender	Male: 23 (27,1%)	Male: 64 (60,4%)	*p* < 0,001
	Female: 62 (72,9%)	Female: 42 (39,6%)	
Smoking status	Smokers: 6 (7,1%)	Smokers: 21 (19,8%)	*p* < 0,001
	Exsmokers: 5 (5,9%)	Exsmokers: 31 (29,2%)	
	Nonsmokers: 74 (87,1%)	Nonsmokers: 54 (50,9%)	
Diabetes mellitus	Yes: 8 (9,4%)	Yes: 28 (26,4%)	*p* = 0,005
	No: 77 (90,6%)	No: 78 (73,6%)

**Table 3:** General characteristics - PTP 1 vs. PTP 2 subgroup

Variable	PTP 1 subgroup	PTP 2 subgroup	Difference sig. (2-tailed)
Age (years)	60,52 ± 8,13	64,84 ± 8,19	*p* = 0,011
Waist circumference (cm)	105,8 ± 11,3	110,4 ± 10,4	*p* = 0,047
Hip circumference (cm)	107,5 ± 9,8	106 ± 9,5	*p* = 0,197
Waist to hip ratio	0,980 ± 0,054	1047 ± 0,068	*p* < 0,001
Body mass index (kg/m^2^)	30,02 ± 5,05	30,17 ± 3,68	*p* = 0,705
LVEF (%)	56,1 ± 8	56,9 ± 7	*p* = 0,618
LVEDd (mm)	51,7 ± 5,9	51 ± 5,5	*p* = 0,771
IVS (mm)	11 ± 1,6	11,8 ± 1,75	*p* = 0,01
LDL (mmol/l)	2,65 ± 0,96	2,53 ± 0,86	*p* = 0,560
HDL (mmol/l)	1,33 ± 0,32	1,29 ± 0,33	*p* = 0,445
TG (mmol/l)	1,67 ± 0,97	1,86 ± 1,11	*p* = 0,312
TCH (mmol/l)	4,65 ± 1,13	4,82 ± 1	*p* = 0,442
eGFR (ml/min/1.73m^2^)	69,8 ± 12,3	73,2 ± 19,9	*p* = 0,442
Vaspin (ng/ml)	0,4495 ± 0,7935	0,1995 ± 0,2187	*p* = 0,019

*LVEF* Left ventricular ejection fraction, *LVEDd* Left ventricular end-diastolic diameter, *IVS* Interventricular septal thickness, *LDL* Low-density lipoprotein, *HDL* High-density lipoprotein, *TG* Triglyceride, *TCH* Total cholesterol, *eGFR* Estimated glomerular filtration rate, *PTP* Pre-test probability

**Table 4:** Nominal variables characteristics - PTP 1 vs. PTP 2 subgroup

Variable	PTP 1 subgroup	PTP 2 subgroup	Difference sig. (2-tailed)
Gender	Male: 31 (44,9%)	Male: 33 (89,2%)	*p* < 0,001
	Female: 38 (55,1%)	Female: 4 (10,8%)	
Smoking status	Smokers: 13 (18,8%)	Smokers: 8 (21,6%)	*p* = 0,489
	Ex-smokers: 18 (26,1%)	Ex-smokers: 13 (35,1%)	
	Non-smokers: 38 (55,1%)	Non-smokers: 16 (43,2%)	
Diabetes mellitus	Yes: 18 (26,1%)	Yes: 10 (27%)	*p* = 0,917
	No: 51 (73,9%)	No: 27 (73%)	

*PTP* Pre-test probability

We did not find a statistically significant difference in vaspin level between HSCS positive and HSCS negative subgroup, unless the pre-test probability was taken into account. In the PTP 1 subgroup, HSCS positive patients had higher (0,579 ± 0,898 ng/ml) mean vaspin concentration than non-HSCS (0,379 ± 0,732 ng/ml) patients (t(66) = −2595; *p* = 0,012; d = 0,658; 1-β = 0,850; *n* = 68). In the PTP 1 subgroup, the vaspin difference observed between patients with (median = 0,301) and without HSCS (median = 0,1385), was also affirmed by using native (non–normalised) vaspin values in the test input of the non-parametric Mann-Whitney test (U = 768; z = 3081; *p* = 0,002; *n* = 68). While assessing the statistical tests assumptions, there was one significant outlier discovered in our dataset, which we decided to remove prior t–test and Mann–Whitney tests were run.

In the PTP 1 subgroup, an increase in the number of diseased coronary vessels and increase in the number of significantly stenotic arteries were both associated with higher vaspin concentration (ρ = 0,269; *p* = 0,027; *n* = 68) and (ρ = 0,340; *p* = 0,005; *n* = 68), respectively. In the entire target group and in the PTP 2 subgroup, this correlation was non-significant.

**Fig. 1:**
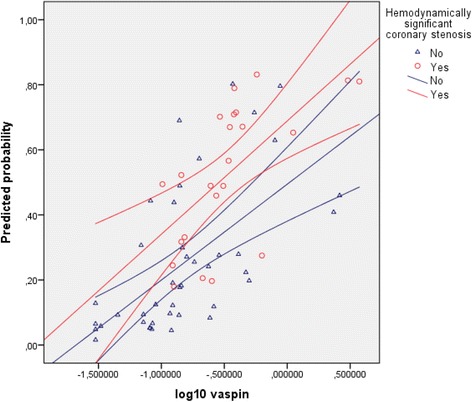
Binomial regression model – the relation between vaspin and HSCS. Legend: The picture depicts regression lines and their 95% confidence intervals. Predicted probability is expressed as a ratio. HSCS: Hemodynamically significant coronary artery stenosis

Binomial regression model was performed to ascertain the effect of age, smoking status, and Log10 vaspin on the result of ICA in PTP 1 subgroup. There were three cases with studentized residuals greater than 2,5 standard deviations in preliminary model. We decided to remove these cases prior the final regression model was constructed. The regression model was statistically significant (χ^2^ = 20,355 (3); *p* < 0,001, *n* = 66). The model explained 36,6% (Nagelkerke R^2^) of the variance in HSCS variable and correctly classified 74,2% of cases. Sensitivity was 52,2%, specificity was 86%, positive predictive value was 66,7%, negative predictive value was 77,1%. The binomial regression model summary is provided in Table 5. The relation between predicted probability of HSCS and Log10 vaspin, including the 95% CI is depicted in Fig. 1.

**Table 5:** Regression model summary - prediction of HSCS based on age, vaspin and smoking

Predictor variable	B coefficient	Standard error	Wald	df	*p*-value	Odds ratio
Age (year)	0,106	0,041	6783	1	0,009	1112
Fasting vaspin (ng/ml)	2168	0,742	8540	1	0,003	8743
Smoking	1599	0,695	5290	1	0,021	4947
Constant	−6266	2574	5929	1	0,015	0,002

*Smoking* smokers compared to non – smokers, *HSCS* Hemodynamically significant coronary artery stenosis

**Fig. 2:**
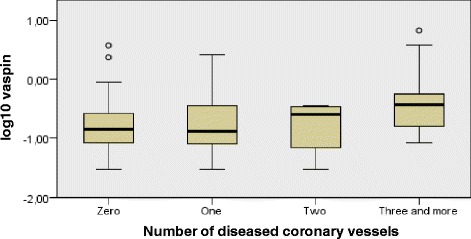
Independent samples Kruskal - Wallis test of vaspin differences in various NDV groups. Legend: The boxplot of log10 vaspin across specific groups of patients differing in the total number of narrowed coronary arteries. PTP: Pre – test probability; NDV: Number of diseased coronary vessels

**Fig. 3:**
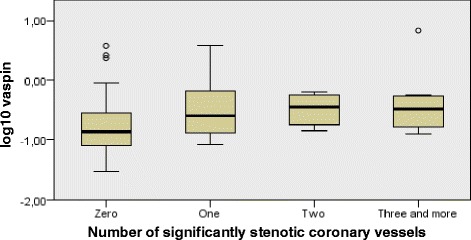
Independent samples Kruskal - Wallis test of vaspin differences in various NrSSV groups. Legend: The boxplot of log10 vaspin across specific groups of patients differing in the number of significantly stenotic coronary arteries. PTP: Pre – test probability; NrSSV: Number of significantly stenotic coronary vessels

Kruskal-Wallis test was conducted to determine if there were differences in vaspin between groups that differed in the number of narrowed coronary arteries in PTP 1 patients: The “zero” (*n* = 30), “one” (*n* = 12), “two” (*n* = 7), “three and more” (*n* = 20) narrowed coronary arteries groups were assessed. Mean vaspin ranks were statistically significantly different between the different “number of narrowed coronary arteries” groups (χ(3) = 8917; *p* = 0,03; *n* = 69). Subsequently, pairwise comparisons were performed using Dunn’s procedure. A Bonferroni correction for multiple comparisons was made. This post hoc analysis revealed statistically significant differences in mean ranks between the “zero” (mean rank = 30,77) and “three and more” (mean rank = 46,25) groups (*p* = 0,045). Boxplot of log10 vaspin medians are depicted in Fig. 2. Similarly, Kruskal-Wallis test was conducted to determine differences in vaspin concentrations between groups that differed in the number of significantly stenotic coronary arteries in PTP 1 patients: The “zero” (*n* = 44), “one” (*n* = 13), “two” (*n* = 4), „three and more “(*n* = 8) groups were assessed. Generally, the distribution of vaspin was significantly different across categories of significantly stenotic arteries groups (χ(3) = 9334; *p* = 0,025; *n* = 69). However, the post hoc Dunn’s analysis revealed no statistically significant differences in mean ranks between various subgroup combinations, when Bonferroni correction was performed. Boxplot of log10 vaspin medians are depicted in Fig. 3. Outside the PTP 1 envelope, we didn’t detect significant differences in vaspin concentrations between various groups of patients divided on the basis of the number of narrowed coronary arteries, nor on the basis of the number of significantly stenotic arteries.

## Discussion

The PTP estimate is currently used as one of the steps taken in the management of SCAD patients in most European countries. Variables entering the PTP estimate include the age, gender and clinical features of coronary artery disease described as one of the following: non–anginal pain, atypical angina, typical angina. According to ESC 2013 Guidelines on management of SCAD patients, the event risk stratification should be performed whenever the estimated PTP is higher than 15%, with subsequent ICA being performed in the high-risk group of patients. A trial of OMT should be performed in low- and intermediate-risk patients. In the high-risk group of patients, the ICA is warranted without the need to perform the OMT trial in advance. In daily clinical practice, there is a grey zone represented as estimated PTP from 15% to 65%, where performing the ICA often fails to reveal any hemodynamically significant coronary artery stenosis. In our cohort, HSCS was found only in 36,2% of ICA patients in the PTP 1 subgroup. In this context, we might conclude that in 63,8% of these patients, coronary angiography was, in fact, unnecessary invasive procedure, if only reassuring the patient and clinician about patent coronary arteries is not considered the main goal of the ICA procedure. Large proportion of patients with negative result of diagnostic ICA among patients with intermediate PTP is problematic not only from medical point of view (possible complications of invasive procedure), but also in respect to the ever-rising health care spending. In daily clinical practice, there is enough space for apprehension-biased decision-making, when it comes to the problem of ICA indication. Despite postponing the ICA in low- to medium-risk SCAD patients is fully warranted according to current ESC Guidelines on SCAD management, theoretical possibility of health complications in these situations often leads to hasty decisions. In our study, we didn’t find significant difference in mean vaspin concentrations between control group and target group. However, we found higher vaspin concentrations in HSCS patients within estimated PTP between 15% and 65%. This result indicates a potential for vaspin to become one of the variables entering the ICA indication algorithm in SCAD patients within the grey zone pre-test probability. This study also demonstrated significant vaspin – BMI correlation and vaspin - WC correlation only in diabetes mellitus SCAD patients, not in non-diabetics. This finding could be, to some extent, explained by the existence of the compensatory effect of vaspin in the situation of increased insulin resistance, which is mediated through the inactivation of human kalikrein 7 [14].

When we divided our test subjects into subgroups according to the estimated PTP, we observed higher plasma vaspin concentration in patients in the PTP 1 subgroup than in the PTP 2 subgroup. This could possibly reflect the protective role of vaspin in pathogenesis of the atherosclerosis, which was recently described by Sun et al. [15]. The protective role of vaspin on the endothelial progenitor cells could be also mediated throughout the insulin resistance-lowering effect of vaspin. If higher estimated PTP represents higher likelihood of CAD presence, significantly lower mean vaspin concentration found in the PTP 2 subgroup could substantiate metabolic effect of vaspin in SCAD patients. On the other hand, the association of higher mean vaspin concentration with lower PTP could reflect the active phase of vascular reparatory and metabolic compensatory effects exerted by vaspin earlier in the course of the disease. Indeed, the progression of CAD is also a function of time, as it is in the case of PTP estimate. Unlike previously observed by Li et al. [9], our data exhibit positive correlation between vaspin and the number of diseased vessels and the number of significantly stenotic vessels. However, this association present in PTP 1 subgroup, was non-existent in the PTP 2 subgroup of patients. Since the PTP percentage positively correlates with the PTP estimate grouping, patients within lower PTP subgroup differ significantly from the general CAD population by the means of the relation between the vascular reparatory effects exerted by vaspin and the extent of the coronary atherosclerosis represented by the number of diseased coronary vessels and/or number of significantly stenotic coronary vessels. In other words, our attitude, by dividing the patients into subgroups according to the PTP, reflects also the role of the time factor in the pathogenesis of coronary artery disease, which was not the case in the study performed by Li et al.

We analysed the connection between the extent of the CAD and the vaspin concentration in the PTP 1 subgroup using the Kruskal-Wallis test. Our analysis doesn’t substantiate the hypothesis of simple-fashioned linear relationship between reparatory/protective effects exerted by vaspin and the extent of CAD, even in the situation of near-constant PTP. The interplay between the vascular reparatory and metabolic protective effects exerted by vaspin seems to exhibit characteristics of a compensatory response, triggered by the progression in the extent of coronary atherosclerosis (Fig. 2). On the other hand, Kruskal-Wallis test revealed, that in terms of HSCS detection, vaspin mean ranks are generally higher in the presence of hemodynamically significant coronary artery stenosis. However, the post hoc analysis didn’t detect significant vaspin differences between the various levels of CAD’s extent represented by the number of NrSSV (Fig. 3). A much larger cohort of patients is needed to elucidate the connection between vaspin concentration and the extent of coronary atherosclerosis, particularly in term of insufficient statistical strength provided by our Dunn’s between-group post hoc analysis.

Further basic research is necessary to describe the time frame of general metabolic and local protective vaspin effects in the process of CAD pathogenesis. Further clinical research is needed to clarify the temporal association between the protective effects exerted by vaspin, the duration of CAD symptomatology and the level of coronary artery narrowing diagnosed by the means of the ICA.

## Study limitations

Larger study allowing us to match the target group against the control group with respect to investigated variables could filter the influence of various confounders, particularly in connection to observed lack of mean vaspin difference between target group and control group. Target group and controls were not matched particularly with respect to gender, smoking status, eGFR and some of the biometric parameters. Therefore, the effect of these possible confounders was not evaluated which, had it been the contrary, could had possibly led us to the opposite finding, that vaspin could be used as a diagnostic marker of CAD. With respect to this drawback, the lack of significant vaspin difference between target group and control group only warrants the conclusion, that there is a lack of evidence that vaspin could be used as an independent surrogate marker for the presence of CAD in general population. On the other hand, the study size is too small to properly evaluate the effect of all possible confounders. Among the other confounders, therapy, dietary modification, physical activity of the patients together with the presence of specific polymorphisms, could have modified vaspin serum concentrations. These confounders were not measured in our study.

The limiting factor is also the possibility that some of the control group subjects might have suffered from clinically silent form of CAD. This could have affected our findings greatly.

In our study, we did not evaluate the intensity of therapeutic control of diabetes mellitus, hypertension, dyslipidaemia and their effect on vaspin concentration, nor we did not address the compliance with the therapy in these comorbidities. Another drawback is the utilisation of FFR technique in current study, which was limited only to few borderline cases.

## Conclusions

Plasma vaspin concentration in SCAD patients probably reflects metabolic compensatory and endothelial reparation processes and differs according to the estimated PTP and the presence of HSCS. Our results indicate, that measuring vaspin in SCAD patients might be clinically useful in patients with PTP below 66%. Evidence that we gathered doesn’t advocate the use of vaspin measurement in the role of a biochemical marker for CAD diagnosis in general population.

## Abbreviations

CAD: Coronary artery disease
CI: Confidence interval
CRP: C – reactive protein
eGFR: Estimated glomerular filtration rate
ELISA: Enzyme-linked immunosorbent assay
FFR: Fractional flow reserve
GRP 78: Glucose - regulated protein 78
HDL: High density lipoprotein
HSCS: Hemodynamically significant coronary artery stenosis
ICA: Invasive coronary assessment
IVS: Interventricular septal thickness
LAD: Left anterior descending
LDL: Low density lipoprotein
LVEDd: Left ventricular end-diastolic diameter
LVEF: Left ventricular ejection fraction
NDV: Number of diseased coronary vessels
NrSSV: Number of significantly stenotic coronary vessels
OMT: Optimal medical therapy
PTP: Pre – test probability
RPM: Revolutions per minute
SCAD: Stable coronary artery disease
TCH: Total cholesterol
TG: Triglyceride
WC: Waist circumference
